# Outcomes from a large 10 year hepatitis C treatment programme in people who inject drugs: No effect of recent or former injecting drug use on treatment adherence or therapeutic response

**DOI:** 10.1371/journal.pone.0178398

**Published:** 2017-06-21

**Authors:** Omar Elsherif, Ciaran Bannan, Shay Keating, Susan McKiernan, Colm Bergin, Suzanne Norris

**Affiliations:** 1 Department of Hepatology, St. James’s Hospital, Dublin, Ireland; 2 Department of Genito Urinary Medicine and Infectious Diseases, St. James’s Hospital, Dublin, Ireland; 3 Drug Treatment Centre Board, Dublin, Ireland; 4 School of Medicine, Trinity College, Dublin, Ireland; Cornell University, UNITED STATES

## Abstract

**Background and aims:**

People who inject drugs (PWID) are historically viewed as having “difficult to treat” hepatitis C disease, with perceived inferior treatment adherence and outcomes, and concerns regarding reinfection risk. We evaluated for differences in treatment adherence and response to Peginterferon-alfa-2a/Ribavirin (Peg-IFNα/RBV) in a large urban cohort with and without a history of remote or recent injection drug use.

**Methods:**

Patient data was retrospectively reviewed for 1000 consecutive patients—608 former (no injecting drug use for 6 months of therapy), 85 recent (injecting drug use within 6 months) PWID, and 307 non-drug users who were treated for chronic hepatitis C with Peg-IFNα/RBV. The groups were compared for baseline characteristics, treatment adherence, and outcome.

**Results:**

There was no significant difference in treatment non-adherence between the groups (8.4% in PWID vs 6.8% in non-PWIDs; RR = 1.23, CI 0.76–1.99). The overall SVR rate in PWID (64.2%) was not different from non-PWIDs (60.9%) [RR = 1.05, 95% CI 0.95–1.17]. There was no significant difference in SVR rates between the groups controlling for genotype (48.4% vs 48.4% for genotype 1; 74.9 vs 73.3% for genotype 3). Former and recent PWID had similar adherence rates.

**Conclusions:**

PWID have comparable treatment adherence and SVR rates when compared to non-drug users treated with Peg-IFNα/RBV. These data support a public health strategy of HCV treatment and eradication in PWID in the DAA era.

## Introduction

People who inject drugs (PWID) represent the majority of the hepatitis C virus (HCV) epidemic in the developed world [1]. The majority of new infections develop in active PWID, with this group accounting for more than 80% of new infections in high-income countries [2]. Furthermore, an additional large reservoir of infection exists amongst former PWID who remain undiagnosed. Public health initiatives which aim to reduce the burden of HCV and its attendant complications, or more ambitious strategies to target HCV elimination will therefore be unsuccessful unless they include strategies for engagement and retention of PWID in treatment and follow-up.

PWID are a highly marginalised group and HCV treatment uptake remains poor in this group with studies reporting annual treatment rates of less than 1.6 per 100 person-years in a community-based inner city cohort in Canada, with similar rates reported in Australia [3–4]. Data from the UK demonstrates a geographical variation in PWID treatment rates, ranging from 5 to >25 per 1000 PWID, and highlights the opportunity for upscaling treatment in areas of low treatment uptake. [5] Barriers to treatment of PWID relate to both system and patient factors, but also to reluctance among providers who perceive this group as “difficult to treat”.

Historically, HCV treatment guidelines have excluded PWID from consideration for treatment because of concerns regarding compliance and re-infection. These concerns have not been realised in several small studies assessing treatment for PWID, and outcomes have been comparable to large randomised trials of dual therapy in non-drug users [6–9]. The American Association for the Study of the Liver (AASLD) guidelines recommend that HCV therapy be considered in persons actively using illicit drugs or on opiate substitution therapy provided they wish to be treated and are willing to maintain frequent monitoring [10]. The recently updated European Association for the Study of the Liver (EASL) recommendations on treatment of HCV advocate treatment for individuals with ongoing risk of infection including active PWID, provided they wish to receive treatment and are willing to maintain regular appointments [11].

Important factors that have limited treatment uptake in PWID are the contraindications and adverse effects of Interferon (IFN) based therapy. A study of PWID in a UK prison outreach clinic found only 15% of HCV RNA positive inmates to be eligible for treatment, with only 50% of eligible patients receiving treatment [12]. The development and availability of IFN-free direct acting antiviral (DAA) regimes with high efficacy, improved tolerability and a limited side effect profile will significantly increase the proportion of patients who can be offered HCV therapy [13–15]. However, adherence to therapy in “real world” population groups will remain paramount in the DAA era to realise the SVR rates observed in clinic trials, as well as limiting the emergence of antiviral resistance. In a recent IFN-free DAA trial in patients with cirrhosis, those with a history of injecting drug use were found to have reduced likelihood of sustained viral response (SVR) [16]. We investigated for differences in HCV treatment adherence and outcome between former PWID, recent PWID and non-drug users treated with IFN and ribavirin.

## Methods

Differences between PWID and non-drug users were analysed for adherence to treatment and outcome in all patients treated for chronic HCV infection in a university teaching hospital from 2002–2012. Anonymised patient data was retrospectively reviewed for the treatment period and monitored for at least 6 months follow-up after treatment. The PWID group also included former and recent drug users who were treated in a community based drug treatment centre. Former PWID was defined as having stopped drug use for 6 months prior to treatment, whereas recent PWID was defined as drug use in the 6 months leading up to treatment. Former PWID did not have routine urinary drug testing during treatment, and abstinence was assessed from patient self-reporting alone. Recent PWID provided weekly urine samples for drug testing on treatment.

### Patients

All patients who received treatment had compensated chronic HCV infection and detectable HCV RNA pre-treatment. Patients with cirrhosis were screened for hepatocellular carcinoma prior to treatment with combined liver ultrasonography and alfafetoprotein. All patients treated over the 10 year period were included in the final analysis. This retrospective audit of clinical outcomes was performed in accordance with the Royal College of Physicians of Ireland guidance on clinical audit. As data controller, the St. James’s Hospital review board approved this retrospective audit of anonymised patient data.

Patients co-infected with HIV were considered for HCV treatment once they were established on an effective antiretroviral regimen or they had evidence of a satisfactory CD4 count (>350 cells/mm^3^) prior to initiation of treatment.

Exclusion criteria for HCV treatment included active alcohol abuse at the time of screening, decompensated cirrhosis, untreated psychiatric conditions, a Haemoglobin of less than 12g/dl at baseline. Relative contraindications to treatment were a neutrophil count of less than 1500/mm^3^, and a platelet count of less than 50 x 10^9^/ mm^3^, although a small number of patients (n = 10) with HIV co-infection with moderate neutropenia and/or a platelet count of 25-50x10^9^ were treated at the discretion of the treating physician. Risk factors for HCV transmission were recorded from the patient history and referral source at the screening assessment visit. Information on illicit or non-prescription drug use was collected directly from patients. The non-PWID group’s risk factors included receipt of infected blood products, sexual transmission, or birth in an area of high HCV prevalence. Alcohol abuse was defined as the consumption of more than 21 standard units per week for men and 14 units per week for women.

### Treatment protocol

All patients received comprehensive psychological assessment by specialised treatment nurses prior to therapy. Patients received standard dose weekly Peginterferon-α subcutaneous injections and weight based Ribavirin for 24 or 48 weeks based on genotype and the presence of pre-existing cirrhosis. Individuals who were HCV RNA positive by polymerase chain reaction (PCR) at week 24 were defined as non-responders and treatment was discontinued. Anaemia was managed by Ribavirin dose reduction and/or the use of Erythropoietin at the discretion of the treating physician. HCV RNA was measured using a highly sensitive serum PCR test (HCV Versant 3.0 Assay, Roche COBAS Taqman or Abbott Real Ti*m*e II HCV assay). Patients treated after 2006 were routinely assessed for rapid virologic response (undetectable HCV RNA after 4 weeks of therapy).

### Outcome measures

A sustained viral response (SVR) was defined as undetectable HCV RNA in serum at 24 weeks follow-up after completion of therapy. Treatment adherence was determined as patients who attended up to the pre-defined treatment completion date, and/or met virological stopping rules for non-response. Non-response was defined as ≤ 2 log decline in HCV RNA by treatment week 12, and/or detectable HCV RNA in serum after 24 weeks of therapy. Relapse was defined as patients with an end of treatment response (HCV PCR undetectable) who did not achieve an SVR. Re-infection was defined as any patient who achieved SVR_24_, but had detectable HCV RNA during longer term follow-up. The classification of reinfection included patients who achieved an end of treatment response, but were diagnosed with a different HCV genotype during 24 weeks of follow-up.

### Analysis

SPSS (Version 21, IBM, Chicago, Illinois, USA) was used for calculations. Chi-squared test for independence and the independent sample t-Test were used to compare baseline characteristics between the PWID and non-PWID groups. The proportions of non-adherence and treatment response between groups were compared and expressed as relative risks (RRs) with a calculated 95% confidence interval. The available number of patients was sufficient to significantly identify a RR of 1.5 between PWID and non-PWID groups with a power of 99% (α = 0.05).

## Results

From January 2002 to December 2012, one thousand patients were treated for chronic HCV infection. Of these, 608 were former PWID, and 85 were recent PWID. These groups were compared with 307 non-drug users who had other defined risk factors for HCV. These risk factors included receipt of infected blood or blood products (clotting factors, anti-D), sexual transmission and birth in an area of high HCV prevalence.

### Baseline patient characteristics

Baseline characteristic for both groups are outlined in Table 1. The majority of former PWID and recent PWID were male, and were significantly younger than the non-PWID group. More than half of former PWID were infected with genotype 3 (52.5%), with genotype 1 infection being the second most common (41.8%). Only 5.6% had a genotype other than 1 and 3. In all, 12% of former PWID had underlying cirrhosis, and 114 (16.5%) were co-infected with HIV.

**Table 1:** Baseline patient characteristics of former and recent PWID and non-PWIDs treated for chronic HCV infection.

	Former PWID (n = 608)	Recent PWID (n = 85)	non-PWID (n = 307)
Age (years) [mean ± SD]	36.2 ± 7.7	35.9 ± 6.6	43.0 ± 11.6
Sex (male) [n (%)]	547 (78.9)	67 (78.8)	196 (63.8)
Genotype [n (%)]			
1	290 (41.8)	42 (49.4)	142 (46.3)
2	29 (4.2)	5 (5.9)	15 (4.9)
3	364 (52.5)	38 (44.7)	131 (42.7)
4–6	10 (1.4)	0 (0)	19 (6.3)
Cirrhosis [n (%)]	83 (12)	0 (0)	43 (14)
HIV Co-infection	114 (16.5)	0 (0)	43 (14)
Log_10_ Viral Load–mean (range)	6.53 (2.10–7.84)	6.63 (4.30–7.33)	6.49 (4.06–7.64)

Of the 85 recent PWID treated for HCV in a community based drug treatment centre during the study period, all received opiate substitution therapy. Genotype 1 infection was more common with lower degrees of fibrosis in the recent PWID group. None of the patients had established cirrhosis.

In the non-PWID group, there were more women (36.2%), and the mean age was significantly higher at 43.0 years. Genotype 1 infection was the most common genotype (46.3%) followed by genotype 3 (42.7%). The proportion of patients with cirrhosis was similar to the former PWID group (14%), as was the percentage with HIV co-infection (14%).

Pre-treatment HCV viral load was available in 557 (91.6%) of former PWID, 85 (100%) of recent PWID and 253 (82.4%) of non-PWID, and a higher percentage of PWID had a viral load of greater than 10^6^ IU/ml.

### Treatment completion/compliance

Six hundred and eight (608) former PWID, 85 recent PWID, and 307 non-PWIDs were commenced on HCV therapy. Treatment was discontinued in 71 former PWID (11.5%), 8 recent PWID (9.4%), and 46 non-PWIDs (15%) for virologic failure at week 24 of therapy. There was no significant difference in treatment non-completion (for reasons other than virologic non-response) between PWID and non-PWIDs [8.4% vs 6.8%, RR = 1.23, 95% CI 0.76–1.99]. Additionally, there was no significant difference in treatment non-completion between former and recent PWID [8.7% vs 5.9%, RR = 0.84, 95% CI 0.33–2.10].

Fifteen patients (17.6%) in the recent PWID group tested positive for opiates at least once during treatment, 11 (12.9%) tested positive for benzodiazepines, and 5 (5.8%) tested positive for cocaine. Seven patients tested positive for two of the drug classes, while 5 tested positive for all three classes. No patients reported injecting illicit drugs during treatment or in the 6 month post-treatment follow-up period.

### Response to treatment

**Fig 1:**
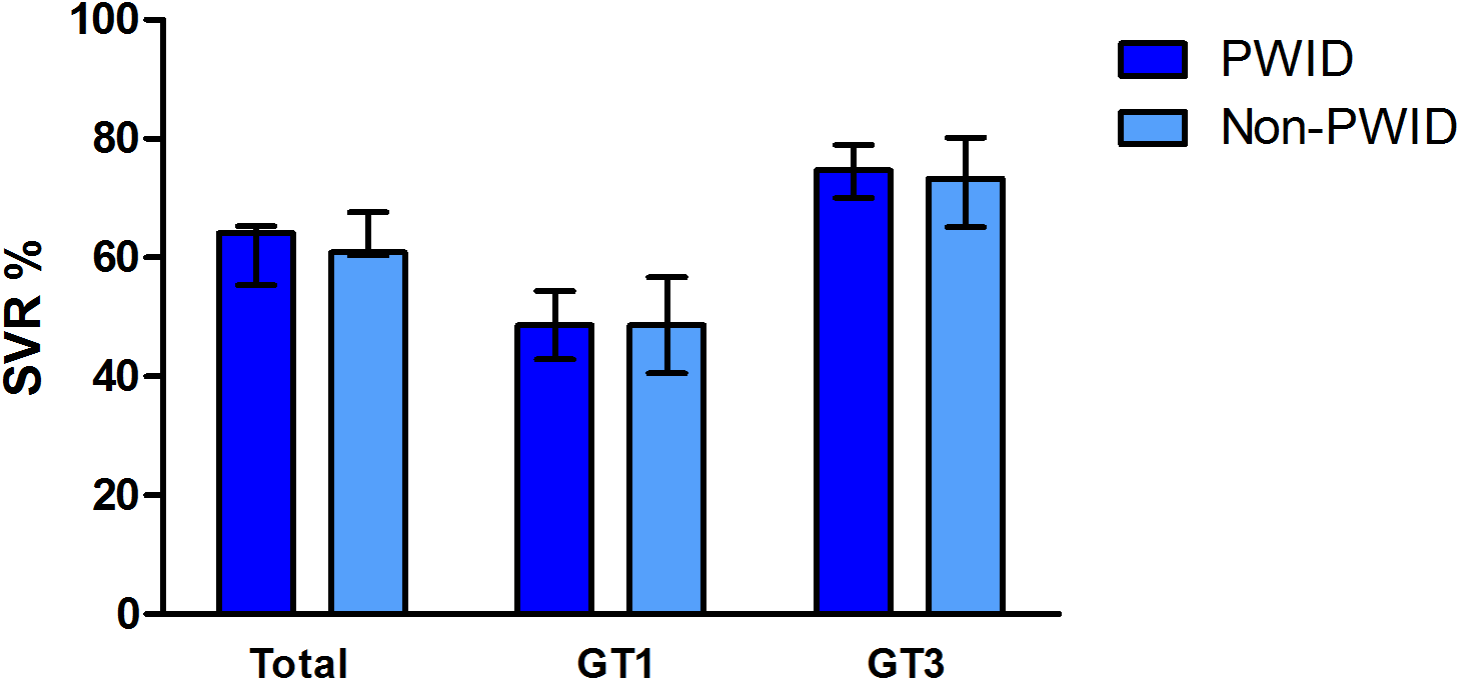
Treatment response. Rates of sustained viral response (95% CI bars) with dual therapy in PWID (people who inject drugs) and non-PWID (a) for all genotypes– 64.1% ss 60.9% (b) genotype 1–48.6% vs 48.6% and (c) genotype 3–74.7% vs 73.3%.

Treatment response rates are detailed in Table 2. The overall SVR rate in PWID (64.1%) was not different from non-PWIDs (60.9%) [RR = 1.05, 95% CI 0.95–1.17]. There was no significant difference in SVR rates between the groups when comparing genotype 1 and genotype 3 infections (Fig 1). As expected genotype 1 infection was less responsive to Interferon therapy in both PWID and non-PWIDs (47.7% versus 48.4%, *p* = non-significant). PWID were more likely to be lost to follow-up after achieving a viral response at the end of treatment [RR = 2.73, 95% CI 1.16–6.43]. There was no significant difference in SVR between former and recent PWID for genotype 1 [47.2% vs 54.8%, RR = 0.82, 95% CI 0.61–1.10] and genotype 3 infection [73.9% vs 83.8%, RR = 0.91, 95% CI 0.77–1.07].

**Table 2:** Adherence and treatment responses in people who inject drugs (PWID) and non-PWIDs.

	Former PWID n (%)	Recent PWID n (%)	Non-PWIDs n (%)	RR (95% CI) in Former versus recent PWID	RR (95% CI) in PWID versus non-PWID
Non-responders	71/608 (11.7)	8/85 (9.4)	46/307 (15)	1.24 (0.62–2.49)	0.74 (0.53–1.05)
Genotype 1 patients	52/248 (21)	8/40 (19)	32/142 (22.5)	1.02 (0.53–1.98)	0.92 (0.63–1.35)
Genotype 3 patients	14/326 (4.3)	0 (0)	7/131 (5.3)	-	0.72 (0.30–1.75)
End of treatment response	454/608 (74.7)	68/85 (82.9)	234/307 (76.2)	0.93 (0.83–1.05)	0.99 (0.92–1.07)
Genotype 1 patients	155/248 (62.5)	28/40 (70)	98/142 (69)	0.78 (0.68–0.90)	0.92 (0.80–1.05)
Genotype 3 patients	270/326 (82.8)	35/38 (94.6)	111/131 (84.7)	0.90 (0.81–0.99)	0.99 (0.91–1.08)
Sustained Viral Response	384/608 (63.2)	60/85 (70.6)	187/307 (60.9)	0.89 (0.77–1.04)	1.05 (0.95–1.17)
Genotype 1 patients	117/248 (47.2)	23/40 (54.8)	69/142 (48.6)	0.82 (0.61–1.10)	1.00 (0.81–1.23)
Genotype 3 patients	241/326 (73.9)	31/38 (83.8)	96/131 (73.3)	0.91 (0.77–1.07)	1.02 (0.91–1.15)
Non-adherence	53/608 (8.7)	5/85 (5.9)	21/307 (6.8)	0.84 (0.33–2.10)	1.23 (0.76–1.99)
Lost to Follow up	30/608 (4.9)	1/85 (1.2)	6/307 (2)	4.19 (0.58–30.4)	2.3 (0.97–5.45)

Follow up data on 219 former PWID with who achieved SVR between 2002–2007 for a median of 57 months (range 6–168 months) indicated that 13 patients were re-infected with HCV, a reinfection rate of 10.5/1000 person years of follow-up. All 13 patients had a relapse in injecting drug use. There was no significant difference in re-infection rate between former PWID with and without HIV co-infection.

## Discussion

This large retrospective study of a decade of HCV treatment outcomes demonstrates that PWID have similar treatment adherence to Peginterferon and Ribavirin as non-PWID patients with chronic HCV infection. Of the 693 recent or former PWID who commenced treatment, over 90% completed treatment at our centre.

Additionally, PWID patients had a comparable response to treatment with an overall SVR rate of 62.4%. More than half of PWID patients were infected with genotypes 2 and 3, which have favourable response rates to interferon-based therapy. Allowing for this, there was no significant difference in SVR observed between the groups when controlling for genotype. PWID patients were younger but this had no effect on the response to therapy.

These data are in line with previously published cohort studies of treatment adherence and outcome in PWID. The Benelux study group reported no effect of a history of drug use on HCV treatment completion [7]. The SVR rate was approximately 40% and did not differ significantly between PWID and non-drug users having controlled for genotype. In the Benelux study, patients received non-pegylated Interferon, and consequently improved response rates were expected with the use of Peginterferon. Recent systematic reviews of HCV (dual therapy) have also illustrated that active PWID can respond favourably to treatment. A high-pooled SVR rate of 55.5% (95% CI 50.6–60.3%) with a high treatment completion rate of 83.4% was found in one systematic review of 11 studies among active PWID [17–18].

HIV co-infection is associated with accelerated progression of liver disease in patients with HCV resulting in increased morbidity and mortality [19–20]. Co-infected patients with a history of injection drug use have an even higher risk for poorer clinical outcomes and consequently have a greater need for therapy. Our co-infected PWID cohort achieved an SVR rate of 60.5%, indicating that this group at risk for unfavourable outcomes has comparable outcomes with the entire cohort.

The accepted consensus just over a decade ago was that PWID should not be offered therapy until they have been drug free for a period of six months [22–23]. Several small studies have since demonstrated that treatment outcomes even in active PWID are non-inferior, which resulted in updated guidelines [6–9]. However, recommendations that active injection drug use should not preclude access to antiviral therapy have not been translated into enhanced treatment uptake among PWID. A study of physicians’ attitudes in Canada found that whilst 90% would consider treating former PWID, only 20% of service providers were likely to treat active or recent drug users [21]. Community based studies of PWID in Australia have shown only a modest improvement in HCV treatment uptake, ranging from 0.5–1.0% in 2004–05 (5–10 per 1000 infected) to 1.5–2.0% in 2009–10 (15–20 per 1000 infected) [24]. The biggest improvements in HCV treatment uptake have been observed in countries that have employed a comprehensive national strategy such as Scotland’s HCV Action Plan [25–26]. Early results have been encouraging in terms of patient attendance at specialist centres following diagnosis and treatment uptake thereafter [27].

Another provider factor that may limit treatment access for PWID is the concern regarding re-infection risk in patients who continue to inject drugs. In our low risk population of former PWID with HCV mono-infection, the re-infection rate was low at 10.5/1000 person years of follow-up, with a median follow-up period of nearly 5 years. As viral homology sequencing was not performed in all patients with detectable HCV RNA after SVR, there was a risk of classifying some late relapse cases as re-infection. This potential classification error may be associated with an overestimation of the re-infection rate. All re-infection cases were associated with a relapse in injection drug use. Uncertainty about re-infection rates in published studies remain, due to high dropout rates in long term follow up. Re-infection rates in a number of studies are consistently low between 3–5% [28–30] with a slightly higher re-infection rate of 6.4% in PWID who reported ongoing injecting drug use post-SVR [17]. A meta-analysis of 43 studies which included 7969 patients treated for HCV demonstrated that rate late-relapse or re-infection is higher in prisoners or those with on-going drug use (22.32/1000 person years of follow-up). The highest risk was in patients with HIV/HCV co-infection (32.02/1000 person years of follow-up). These rates are much higher than we observed in our cohort. Genotyping and viral sequencing was not universally used in classifying late relapse or re-infection in these studies, possibly resulting in an over-estimation of the re-infection rates [31].

A number of factors preclude some HCV infected PWID from successful treatment with existing Interferon-based therapy. Patient related factors which include chaotic lifestyles, psychiatric comorbidity and depression may influence patient adherence during treatment. Patient selection is therefore critical and may be optimised through careful pre-treatment assessment management, with particular attention given to patient and provider factors known to impact on compliance. In our study, a combined clinician and nurse specialist assessment was used to select patients for treatment on a case-by-case basis. Patients with active psychiatric comorbidity were assessed by liaison psychiatry as part of the multi-disciplinary pre-treatment assessment. Whilst this assessment process introduces an element of selection bias, the high observed adherence and SVR rates in PWID result from treatment recruitment decisions made on an individual basis. A multidisciplinary approach to HCV treatment where treatment and counselling services are offered in a “one-stop-shop” has been shown to improve treatment uptake and adherence with therapy [32].

These data support the inclusion of recent and former PWID in any public health HCV treatment strategy aimed at reducing HCV prevalence as a first step towards elimination. The European guidelines advocate early DAA treatment for those with an on-going infection risk including PWID [11]. Interferon free DAA regimens are now the standard of care, with treatment duration as short as 8 weeks, and observed SVR rates of > 95% [13–15]. Co-morbid psychiatric conditions seen in up to 50% of PWIDs will no longer be a contraindication to therapy [33]. As treatment regimens become simpler and more tolerable, this should result in an expansion of the eligible treatment population. With simplification of therapy, it seems paradoxical that many payers have imposed even greater access restrictions to treatment, with some states in the US requiring 12 months abstinence from drug and alcohol use for re-imbursement [34]. These restrictions are difficult to justify in the light of increasing evidence that HCV treatment is effective in PWID. The use of pre-treatment screening tools for illicit drug use do not help in identifying patients more likely to respond to therapy, and only serve as an added barrier to treatment, and are therefore discouraged by treatment guidelines [35]. Some concerns remain regarding the risk of re-infection, but this requires strategies to reduce the re-infection risk as part of treatment strategies rather than excluding PWID altogether. The nature of epidemic control is that re-infection risk will decline by successful treatment scale-up strategies in the populations with the highest prevalence, as the reservoir for re-infection in PWID is reduced. There is a growing body of evidence supported by modelling data of such a “treatment as prevention” paradigm [36]. Additionally, prioritising PWID for treatment appears to be more a cost-effective initiative at reducing long-term health costs that treating non-PWID with comparable degrees of fibrosis [37]. HCV elimination is an ambitious target, but it will not be achieved by excluding PWID from treatment.

## Supporting information

S1 File Anonymised PWID study source data. (XLSX)

## Abbreviations

PWID: People who inject drugs
Peg-IFNα: Pegylated Interferon-α
RBV: Ribavirin
HIV: Human Immunodeficiency virus
SVR: Sustained viral response
DAA: Direct acting antivira
HCV: Hepatitis C virus
